# Supplementary material for: Loss of Human Beta Cell Identity in a Reconstructed Omental Stromal Cell Environment
Source: Cells. 2022 Mar 8;11(6):924. doi: 10.3390/cells11060924 (PMC8946101; doi:10.3390/cells11060924)
Supplement: Supplementary file 1 [file cells-11-00924-s001.zip › cells-1632448-supplementary.pdf]

Patient Number	SEX	AGE	BMI	DIABETIC	Hb (g/dl)	Cholesterol (g/dl)	Hypercholesterolemia	Hypertriglycéridemia	HBP	SAS	Blood Glucose (mg/dl)	HDL (g/l)	LDL (g/l)	GGT (U/L)	HbA1C (%)	AST (U/L)	ALT (U/L)
**110**	F	36	58	NO	14.6	1.51	NO	NO	NO	YES	0.95	0.38	0.95	4	5.62	27	30
**2610**	F	37	44	NO	13	3.22	NO	NO	NO	NO	0.77	0.53	2.36	27	X	16	15
**593**	M	26	50	NO	x	x	NO	NO	NO	YES	x	x	x	x	x	x	x
**595**	F	47	40	NO	13.8	2	NO	NO	NO	NO	1.2	0.59	1.15	X	7	30	41
**2611**	F	46	46	NO	13.3	1.8	NO	NO	NO	NO	X	0.7	0.94	X	5.7	21	16
**601**	F	56	32	NO	14.8	2.46	NO	NO	YES	NO	0.99	0.7	1.65	19	X	13	13
**604**	M	49	42.5	YES	X	X	NO	NO	YES	YES	X	X	X	X	X	X	X
**605**	F	34	40	NO	13.9	2.57	NO	NO	NO	NO	0.91	0.68	0.76	10	4.97	15	12
**606**	M	72	47	YES	12.2	X	YES	YES	YES	YES	1.55	X	X	X	X	X	X
**609**	F	20	59	NO	11.5	1.87	NO	NO	NO	NO	1.03	0.46	1.27	X	6.07	X	X
**610**	M	26	54	NO	14.6	1.81	NO	NO	NO	NO	1.02	0.41	1.17	56	5.6	43	65
**611**	F	32	47	YES	13.7	2.06	NO	NO	NO	NO	2	0.39	1.21	48	9.44	13	23
**613**	F	40	41	YES	10.2	X	NO	NO	YES	NO	1.2	X	X	X	7.7	X	X
**614**	F	23	39	NO	14.6	1.39	NO	NO	NO	NO	X	0.43	0.12	11	5	20	16
**617**	F	37	50	NO	13.3	1.82	NO	NO	NO	NO	0.89	0.35	1.16	19	5.6	28	50
**618**	F	36	39	NO	13.6	1.71	NO	NO	NO	NO	X	0.56	0.93	25	5	13	16
**619**	M	53	35.6	YES	14.9	1.97	NO	YES	YES	YES	1.28	0.32	0.97	110	6.1	20	40
**1511**	F	54	43	NO	12.9	1.74	NO	NO	YES	NO		0.44	0.96	26	5.7	21	24
**1611**	F	55	38	NO	X	1.13	NO	NO	YES	NO	X	X	X	21	X	25	29
**1811**	F	41	38.6	NO	13	2.56	NO	NO	NO	NO	0.9	0.68	1.77	X	5.47	18	19
**1001**	F	42	37	X	15	X	X	X	YES	X	X	X	X	X	X	X	X
**1701**	F	47	39	NO	X	X	NO	NO	NO	NO	X	X	X	X	X	X	X
**1801**	F	40	35	NO	X	X	NO	NO	NO	NO	X	X	X	X	X	X	X
**2401**	F	52	35	YES	12.3	X	NO	NO	NO	NO	0.82	X	X	X	X	X	X
**2501**	M	31	52	NO	X	X	NO	NO	NO	YES	X	X	X	X	X	X	X
**702**	F	28	40	NO	13.9	1.26	NO	NO	NO	NO	0.89	0.38	0.79	10	5	12	16

Supplementary Table S1. Clinical characteristics of human donors.

Primer	Forward	Reverse
CYCLO	ATGGCAAATGCTGGACCCAACA	ACATGCTTGCCATCCAACCACT
AP2	AAGAGAAAACGAGAGGATGA	CACAGAATGTTGTAGAGTTCA
IL6	ATAGGACTGGAGATGTCTGAGG	GCTTGTGGAGAAGGAGTTCATAG
TGFB1	ATACAGCAACAATTCCTGGCG	GATGTCCACTTGCAGTGTGT
MAFA	ATTCTGGAGAGCGAGAAGTGCCAA	CGCCAGCTTCTCGTATTTCTCCTT
NKX6-1	GAAGAGGACGACGACTACAATAAG	CTGCTGGACTTGTGCTTCT
PDX1	TACTGGATTGGCGTTGTTTGTGGC	AGGGAGCCTTCCAATGTGTATGGT
PRE INSULIN	TCTACCTAGTGTGCGGGGAA	AGCAATGGGCAGTTGGCTC
IAPP	TTGGTGCCATTCTCTCATCTAC	CAAGTAATTCAGTGGCTCTCTCT
HES1	AGAAAGATAGCTCGCGGCATT	TACTTCCCCAGCACACTTGG
c-MYC	GTAGTGGAAAACCAGCAGCC	AGAAATACGGCTGCACCGAG
SOX9	ACCTATCCAAGCGCATTACCCAC	ATCATCCTCCACGCTTGCTCTGA
PAX4	AATTCCCTGGACTCAGGACTGCTT	TTCCAAGCCATACAGTAGTGGGCA
STAT3	GCTGCACCTGATCACCTTTG	AGGTTCCAATTGGGGGCTTG
STAT1	GGCAAAGAGTGATCAGAAACAA	GTTCAGTGACATTCAGCAACT
SOCS3	GTCCCCCCAGAAGAGCCTATTA	TTGACGGTCTTCCGACAGAGAT
FGB	TTCCTGTGGTGTCTGGCAAA	TGCCAAAGTCAACACTACCGT
IL6ST	TTGTTGGCAAATCAGATGCAG	CCTTTATGGATTCAGGGCTTCC
SREBF1	GCACCCACTCCATTGAAGAT	GGCACTGACTCTTCCTTGATAC
SCD	ACAACTACCACCACTCCTTTC	GGAGACTTTCTTCCGGTCATAG
FASN	TACGACTACGGCCCTCATTT	CCATGAAGCTCACCCAGTTATC
PCSK9	TCCACGCTTCCTGCTGCCAT	CAGGCAGTCAGGGTCCAGCC
LDLR	GAGGTCCACATTTGCGACAAC	GTCATCCTCCAGACTGACCATC
HMGCR	ATTTTGGGTATTGCTGGCCTT	ATGTGCTTGCTCTGGAAAGGT
SMAD7	TGTCCAGATGCTGTGCCTTCCT	CTCGTCTTCTCCTCCCAGTATG
BMP5	TCACCAGCGAAGGCATTACA	AAGCTTGGGCCTTTCTTGTG
SMAD2	GCACCCTGCAACAGTGTGTA	GAGTACTTGTTACCGTCTGCC
SMAD3	GCTTTGAGGCTGTCTACCAGT	ACACACTGGAACAGCGGATG
NFKBIA	TGTCCTTGGGTGCTGATGTC	TCAGCCCCACACTTCAACAG
IRF1	CCCTGGCTAGAGATGCAGATTA	GGCATCCTTGTTGATGTCCCA
RELA	TGAGCCCACAAAGCCTTATC	ACAATGCCAGTGCCATACA
NFKBIZ	CCTAATTCAAATGGGAGCAGCG	GCAGCTCCAAAAAGAGGCGA
C2CD4A	ACGCCAGCCTGATACTCCTA	CCGCCACACCTAGCCTATTA
IL13RA1	GGGAGCCAGCTCAAATTGTA	CCCCACTTGCAGACAAATCC
PARP9	AGCTGGGACAAGAAACCACC	ACTTTGCCACAGGTCCAACT
DTX3L	CAGCACCCAGGAACACGAAG	TACCAGGAAAATGGGCACAGG
JUNB	TTGTCAAAGCCCTGGACGAT	GGTTGGTGTAAACGGGAGGT
PLOD2	ATGGACTTTTGCCGTCAGGAT	TTGGACCACAGCTTTCCATGA
SBNO2	GGCTGCAGTTTGAGGCTCTGA	GCAGGGTTATCGTCCCAGATG

Supplementary Table S2. List of primers.

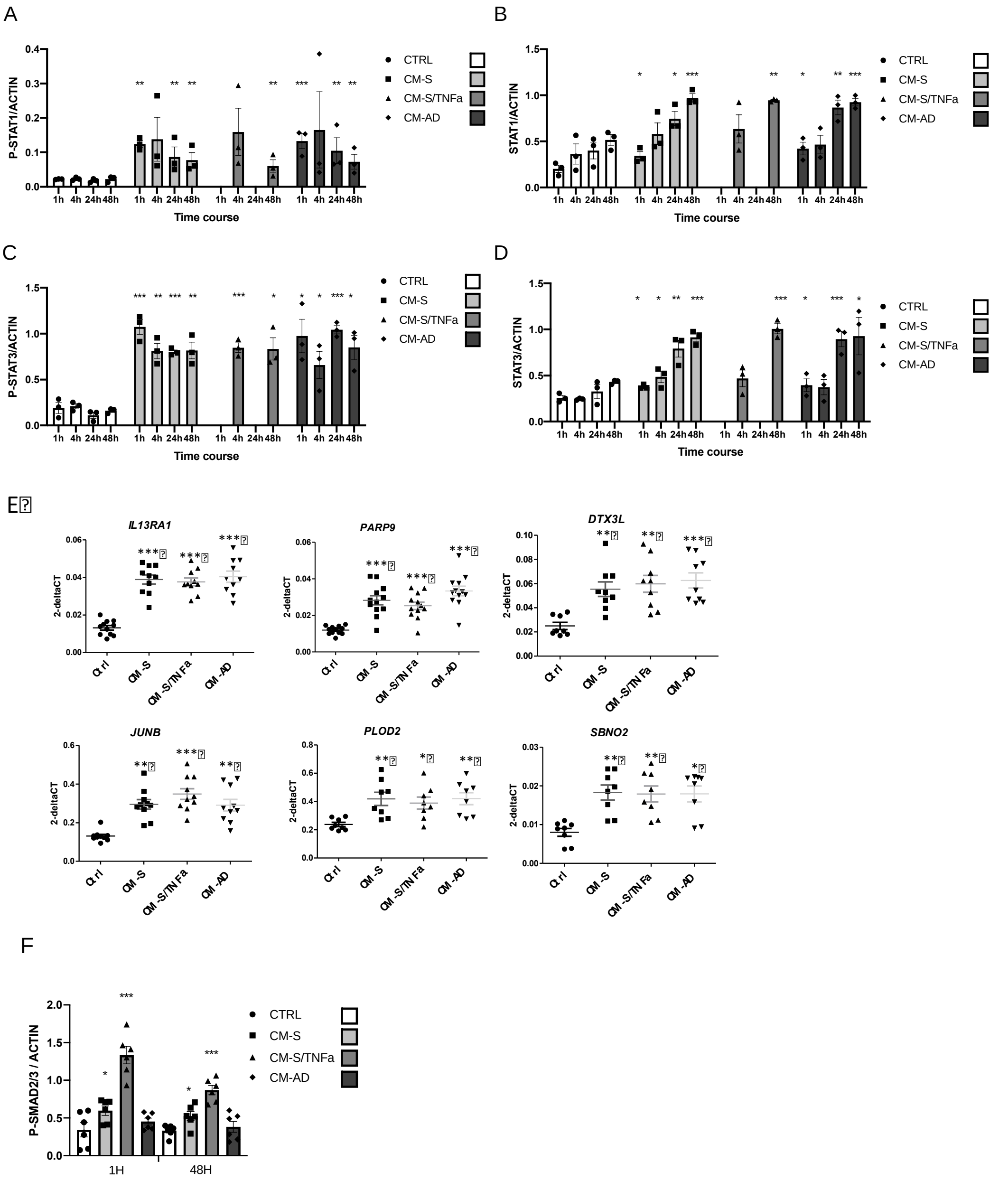

Figure S1. CM from omental-derived cells activates STAT and SMAD signaling in EndoC-βH1 cells. (A-D) Quantification of STAT1 and STAT3 phosphorylation and STAT1 and STAT3 proteins relative to ACTIN. (E) Genes linked to the STAT pathway were analyzed by RT-qPCR after 48H treatment. (F) Quantification of SMAD2/3 phosphorylation relative to ACTIN. The error bars represent the mean ± SEM of at least three separate experiments. *P<0.05; **P<0.01; ***P<0.005.

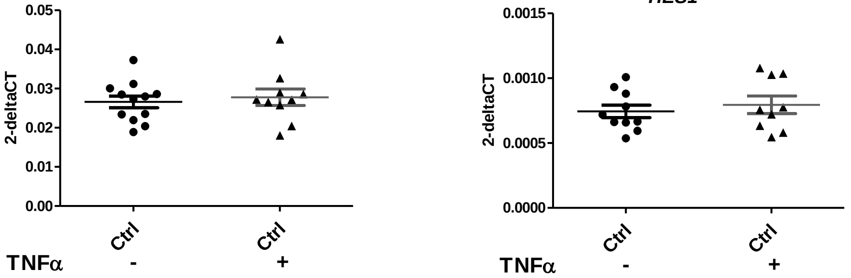

Figure S2: Recombinant TNF-α does not modulate MAFA and HES1 expression in EndoC-βH1 cells. MAFA and HES1 were analyzed by RT-qPCR after 48H treatment. Data are represented as mean ± SD of 5-14 biological replicates. **p < 0.01, ***p < 0.001.

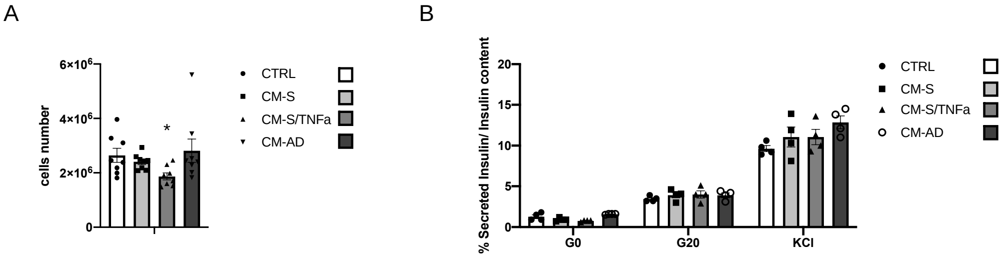

Figure S3: Conditioned media do not modulate beta cell survival or glucose-stimulated insulin secretion. EndoC-βH1 cells were treated with the different conditioned media during 48 h. (A) Cell number quantification (n=8 per condition). (B) Insulin secretion following a 1-hour incubation with glucose or KCl. Secreted insulin is presented as % of content (n = 4).

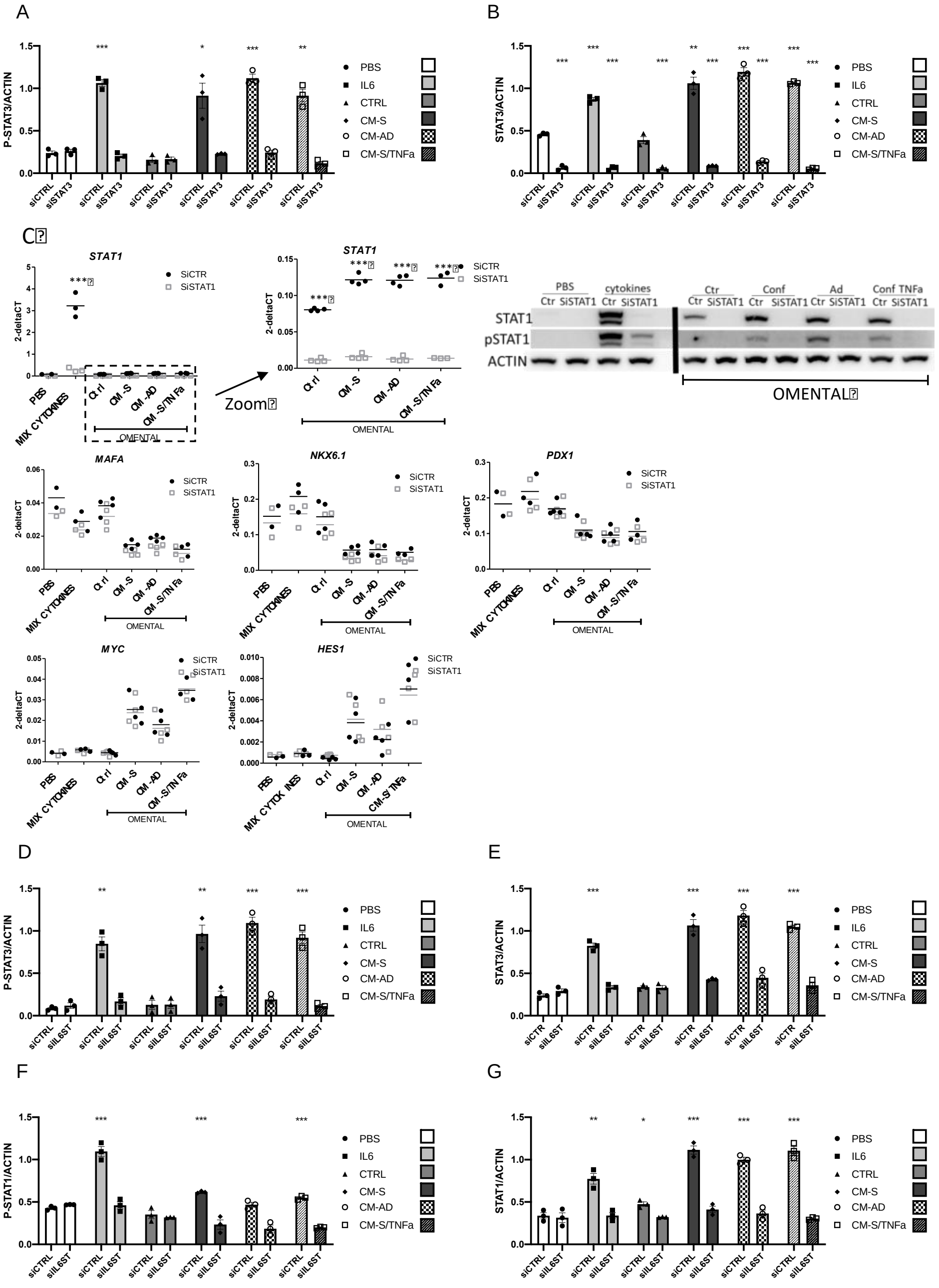

Figure S4: Efficient STAT1, STAT3 or IL6ST knock-down does not revert the effect of CM on beta cell identity. (A-B) EndoC-βH1 were transfected with siRNA targeting STAT3. Quantification of STAT3 phosphorylation and STAT3 proteins are presented relative to ACTIN. The error bars represent the mean ± SEM of three separate experiments. $^{*}P<0.05$; $^{**}P<0.01$; $^{***}P<0.005$. (C) Efficient STAT1 knock-down does not revert the effect of CM on beta cell identity. EndoC-βH1 cells were transfected with control nontarget siRNA (siCTRL) or siRNA targeting STAT1 (siSTAT1). Two days later, cells were treated with either a mix of cytokines (IFNγ and IL1β) or with CMs for 48H. Analyses were performed by either RT-qPCR or Western blot. RTqPCR data are represented as mean ± SEM of 4-6 biological replicates. $^{***}P < 0.001$. (D-G) EndoC-βH1 were transfected siRNA targeting IL6ST. Quantification of STAT1 and STAT3 phosphorylation and STAT1 and STAT3 proteins are presented relative to ACTIN. The error bars represent the mean ± SEM of three separate experiments. $^{*}P<0.05$; $^{**}P<0.01$; $^{***}P<0.005$.

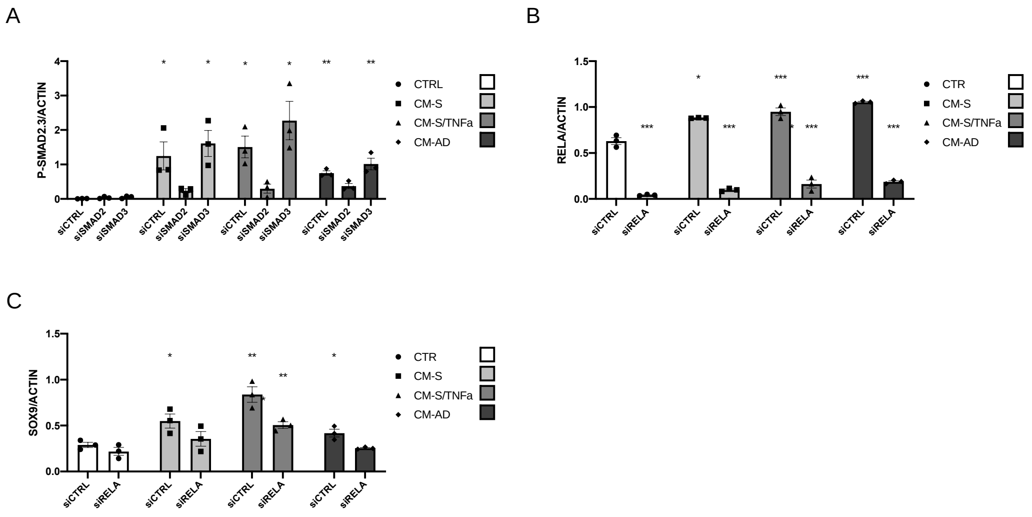

Figure S5: Efficient SMAD2/3 or RELA knock-down does not revert the effect of CM on beta cell identity. EndoC-βH1 were transfected with siRNA targeting SMAD2, SMAD3 or RELA. Quantification of (A) P-SMAD2/3, (B) RELA and (C) SOX9 are presented relative to ACTIN. The error bars represent the mean ± SEM of three separate experiments. *P<0.05; **P<0.01; ***P<0.005.
